# Synthesis of Dimethyl Aryl Acylsulfonium Bromides from Aryl Methyl Ketones in a DMSO-HBr System

**DOI:** 10.3390/molecules181215717

**Published:** 2013-12-16

**Authors:** Zhiling Cao, Dahua Shi, Yingying Qu, Chuanzhou Tao, Weiwei Liu, Guowei Yao

**Affiliations:** 1 Jiangsu Key Laboratory of Marine Biotechnology, Jiangsu Institute of Marine Resources, Huaihai Institute of Technology, Lianyungang 222005, China; 2 School of Chemical Engineering, Huaihai Institute of Technology, Lianyungang 222005, China; 3 School of Life Science, Beijing Institute of Technology, Beijing 100081, China

**Keywords:** dimethyl aryl acylsulfonium, aryl methyl ketones, DMSO-HBr, synthesis, arylglyoxals

## Abstract

A new, simplified method for the synthesis of dimethyl aryl acylsulfonium salts has been developed. A series of dimethyl aryl acylsulfonium bromides were prepared by the reaction of aryl methyl ketones with hydrobromic acid and dimethylsulfoxide (DMSO). This sulfonium salt confirms that bromine production and the bromination reaction take place in the DMSO-HBr oxidation system. What’s more, it is also a key intermediate for the synthesis of arylglyoxals.

## 1. Introduction

In 1957, Kornblum first reported oxidation of α-bromoketones into α-ketoaldehydes by dimethylsulfoxide (DMSO) [1]. Schipper also found that 1,3-diketones could be directly oxidized to 1,2,3-trione derivatives in a DMSO-HBr system [2]. This method has been modified by Folyd and applied to the preparation of aryldiketone (arylglyoxal) derivatives from arylmethyl ketones in high yield [3]. Aryldiketones and arylglyoxals are important building blocks in organic synthesis, particular in the synthesis of biologically active phenylimidazoles, oxazoles, and quinolines [4,5,6,7].

The DMSO-HBr system has been proved to be extremely efficient for the oxidation of the α-methyl group of arylketones [8]. Compared with the traditional selenium dioxide oxidation [9], DMSO-HBr oxidation is milder, less toxic and easy to perform. Furthermore, arylalkynes and alkenes were found to be suitable as substrates for this oxidation system [10,11].

**Scheme 1:**
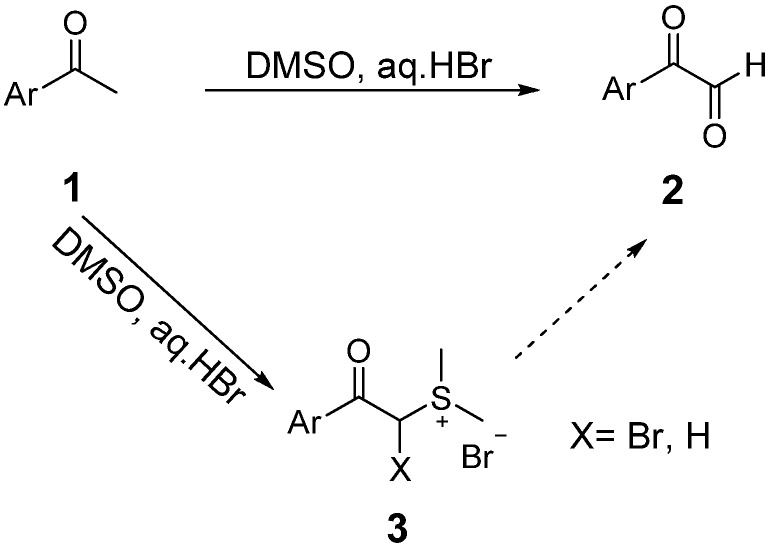
Reaction of aryl methyl ketones in the DMSO-HBr system.

However, when we tried to prepare arylglyoxals **2** from aryl methyl ketones **1** with the DMSO-HBr oxidation system in a sealed flask, we observed that aryl acylsulfonium bromides **3** were unexpectedly produced (Scheme 1). Sulfonium salts, characterized by low sulfur valence and relatively unstable carbon-sulfur bonds, are very useful in practical applications [12]. For example, dimethyl phenacylsulfonium salts can form stable sulfur ylides, which have aroused great interest among researchers [13,14,15]. David recently reported the synthesis of trisubstituted cyclopropanes by condensation of sulfonium ylides with α,β-unsaturated aldehydes [16]. Aryl acylsulfonium bromides can also be used to synthesize 1,4-dicarbonyl compounds by condensation with arylglyoxals [17]. Acylsulfonium bromide salts are normally prepared from dimethyl sulfide and an α-bromoketone. In this study, we report a new and efficient route to aryl acylsulfonium salts and α-bromoacylsulfonium salts from aryl methyl ketones using the DMSO-HBr oxidation system.

Sulfonium salts **3** have never been reported as key intermediate products in DMSO-HBr oxidations of arylmethyl ketones. The formation of salt **3** confirms that the production of bromine and the bromination reaction take place in the DMSO-HBr system. Catalytic oxidation of methyl ketones by vinyl bromide in DMSO is another piece of evidence to support this approach [18]. The mechanism of DMSO-HBr mediated oxidation is still under intense and controversial discussion. Sulfonium salt **3** provides a new basis and reference mechanism for the oxidation of methyl ketones in DMSO-HBr system.

## 2. Results and Discussion

The transformation of aryl acetones **1a**–**h** into sulfonium salts **3a**–**h** in moderate yields (48%–75%, Table 1) was achieved in a mixture of 48% hydrobromic acid (HBr) and DMSO in a sealed flask over 10 h. White solid precipitates could be collected after addition of ether and ethyl acetate to the reaction mixtures. These crystalline solids were readily soluble in water, but insoluble in organic solvents. The ^1^H- and ^13^C-NMR spectra of the products clearly indicated the formation of the sulfonium salt derivatives. Various substituents at the aromatic ring have been shown to affect the yields of the final products. What’s more, in the case of aryl acetones **3e**–**h**, α-bromoacylsulfonium salt products were obtained.

**Table 1:** Synthesis of dimethyl aryl acylsulfonium bromides **3a**–**h**.

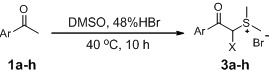			
Entry	Aryl Acetone	Product	Yield (%)
a	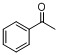	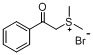	69
b	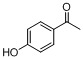	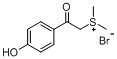	57
c	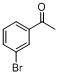	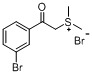	71
d	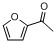	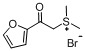	62
e	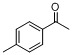	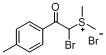	75
f	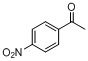	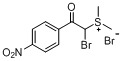	55
g	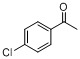	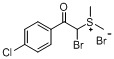	57
h	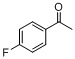	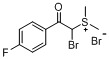	48

Table 2 collects data on the reaction of acetophenone (**1a**) with DMSO-HBr under different experimental conditions. It is revealed that the reaction time and temperature were very crucial for the synthesis of **3**. An increased molar ratio of HBr not only increases the yield, but also shortens the reaction time. We thus obtained the best results using 3 equiv. of HBr (aq 48%) at 40 °C in the presence of DMSO (Table 2, entry 2).

**Scheme 2:**
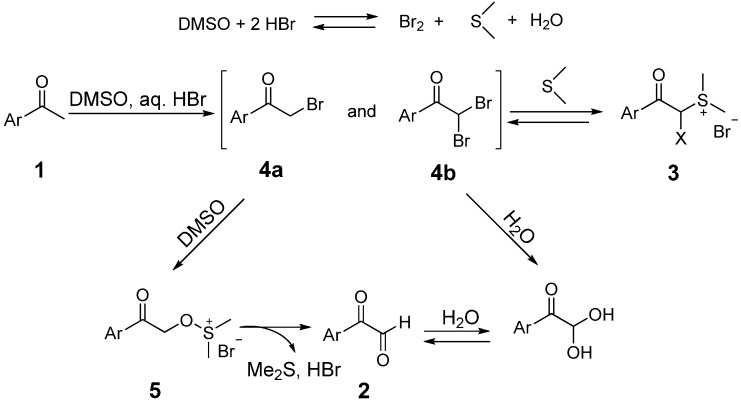
Mechanism for the formation and decomposation of acylsulfonium bromide **3**.

The proposed mechanism for the synthesis of acylsulfonium bromides **3** is shown in Scheme 2. First, oxidation of HBr with DMSO results in the formation of molecular bromine, which reacts with the arylacetone **1** to form the α-bromoketone **4a** and α-dibromoketone **4b**. In addition, dimethyl sulfide is also produced in this oxidation step. The whole reaction is performed in a sealed flask to minimize the escape of dimethyl sulfide which on condensation with bromoketones **4a** and **4b** affords the sulfonium salt **3**.

**Table 2:** Optimization of reaction conditions for synthesis of sulfonium salt **3a** *^a^*.

Entry	Molar Ratio Methyl Ketone/HBr	Time (h)	Temp. (°C)	Yield (%)*^b^*
1	1:3	6	40	41
2	1:3	10	40	69
3	1:3	12	40	56
4	1:3	10	55	45
5	1:1	12	40	12
6	1:5	6	40	61

*^a^* All reactions were heated in a sealed flask containing the same volume of DMSO; *^b^* Isolated yields.

The oxidation of aryl methyl ketones with DMSO is complicated and two mechanisms were proposed recently in the literature. According to the Kornblum reaction, α-bromoketone **4a** is rapidly converted into arylglyoxal **2** through an alkoxydimethyl sulfonium intermediate **5** [8]. Floyd *et al.* have suggested that the hydrolysis and bromination of α-bromoketone **4a** gives an α-hydroxy-α-bromo intermediate, which is hydrolyzed to glyoxal **2** [3]. In the present paper, sulfonium salt **3** exists in the DMSO-HBr oxidation system. The above observations suggest that sulfonium salt **3** is also a key intermediate for synthesis of glyoxal **2**, and it would be decomposed to α-bromoketone **4a** and α-dibromoketone **4b** by heating in an open system. As shown in Scheme 2, both the reaction of **4a** with DMSO and the hydrolysis of **4b** yield the same product **2**. Sulfonium salt **3** is soluble in water and DMSO, and its conversion is very rapid in an open system. In this study, a series of acylsulfonium bromide salts were successfully synthesized by controlling the reaction conditions and precipitation using ether and ethyl acetate.

## 3. Experimental

### 3.1. General

All reagents for synthesis were purchased from TCI Shanghai Co. (Shanghai, China) unless otherwise specified. Melting points were measured on an XT-4 melting point apparatus (Beijing Tech Instrument, Beijing, China) and are uncorrected. NMR spectra were measured using a Bruker AQS AVANCE 300 MHz spectrometer ((Bruker Instruments, Karlsruhe, Germany) with tetramethylsilane as the internal standard. Mass spectra were recorded with an Agilent Technologies MSD SL Trap mass spectrometer (Agilent Technologies, Palo Alto, CA, USA) with an ESI source coupled with an 1100 Series HPLC system. Silica gel GF254 plates (Yantai Chemical Industrials, Yantai, China) was used for thin-layer chromatography (TLC). UV light (λ 254 nm) detection was used.

### 3.2. General Procedure for the Synthesis of Sulfonium Salts **3a–h**

Aryl methyl ketones **1a**–**h** (39.5 mmol) were dissolved in a mixture of 48% hydrobromic acid (20 mL) and dimethylsulfoxide (20 mL) in a sealed flask. This mixture was heated at 40 °C for 10 h and then cooled. After the addition of ethyl acetate (15 mL) and ethyl ether (15 mL), the solution was stirred for another 0.5 h and allowed to stand overnight in the ice box. The precipitate was filtered and washed with ethyl ether to afford the desired sulfonium salt as white crystals.

*Dimethylphenacylsulfonium bromide* (**3a**). Yield: 69%, mp 145–147 °C (lit. [3] 148–152 °C). ^1^H-NMR (DMSO-*d_6_*) δ 8.03 (d, 2H, *J* = 7.5 Hz, Ar-H), 7.8 (t, *J* = 7.4 Hz, 1H, Ar-H), 7.64 (t, *J* = 7.7 Hz, 2H, Ar-H), 5.53 (s, 2H, CH_2_), 2.99 (s, 6H, 2CH_3_); ^13^C-NMR (DMSO-*d_6_*) δ 191.37, 133.96, 135.09, 129.22, 128.70, 52.88, 24.63; MS (ESI) *m/z*: 180.9 [C_10_H_13_OS]^+^.

*Dimethyl-(4-hydroxylphenacyl)sulfonium bromide* (**3b**). Yield: 57%, mp 151–152 °C. ^1^H-NMR (DMSO-*d_6_*) δ 10.83 (s, 1H, OH), 7.90 (d, *J* = 8.7 Hz, 2H, Ar-H), 6.94 (d, *J* = 8.7 Hz, 2H, Ar-H), 5.41 (s, 2H, CH_2_), 2.95 (s, 6H, 2CH_3_); ^13^C-NMR (DMSO-d_6_) δ 189.21, 163.84, 131.58, 125.40, 115.84, 52.68, 24.57. MS (ESI) *m/z*: 197.2 [C_10_H_13_O_2_S]^+^; Anal. calcd. for C_10_H_13_BrO_2_S: C, 43.33; H, 4.73. Found: C, 43.19; H, 4.62.

*Dimethyl-(3-bromophenacyl)sulfonium bromide* (**3c**). Yield: 71%, mp 142–143 °C. ^1^H-NMR (DMSO-*d_6_*) δ 8.20 (s, 1H, Ar-H), 8.01 (t, *J* = 7.8 Hz, 2H, Ar-H), 7.61 (t, *J* = 7.9 Hz, 1H, Ar-H), 5.52 (s, 2H, CH_2_), 2.98 (s, 6H, 2CH_3_); ^13^C-NMR (DMSO-*d_6_*) δ 190.54, 137.53, 136.02, 131.46, 131.31, 127.59, 122.44, 52.73, 24.74. MS (ESI) *m/z*: 259.1 [C_10_H_12_BrOS]^+^; Anal. calcd. for C_10_H_12_Br_2_OS: C, 35.32; H, 3.56. Found: C, 35.25; H, 3.43.

*Dimethyl-(furan-2-acyl)sulfonium bromide* **(3d**). Yield: 62%, mp 132–135 °C. ^1^H-NMR (DMSO-*d_6_*) δ 8.21 (d, *J* = 1.1 Hz, 1H, Ar-H), 7.74 (d, *J* = 3.7 Hz, 1H, Ar-H), 6.87 (dd, *J* = 3.7, 1.6 Hz, 1H, Ar-H), 5.28 (s, 2H, CH_2_), 3.00 (s, 6H, 2CH_3_); ^13^C-NMR (DMSO-*d_6_*) δ 178.64, 149.99, 149.58, 121.96, 113.51, 50.70, 24.70. MS (ESI) *m/z*: 171.2 [C_8_H_11_O_2_S]^+^; Anal. calcd. for C_8_H_11_BrO_2_S: C, 38.26; H, 4.41. Found: C, 38.34; H, 4.58.

*Dimethyl-(a-bromo-4-methylphenacyl)sulfonium bromide* (**3e**). Yield: 75%, mp 144–145 °C. ^1^H-NMR (DMSO-*d_6_*) δ 7.99 (d, *J* = 7.8 Hz, 2H, ArH), 7.86 (s, 1H, CH), 7.47 (d, *J* = 7.8 Hz, 2H, Ar-H), 3.07 (s, 3H, SCH_3_), 3.01 (s, 3H, SCH_3_), 2.44 (s, 3H, Ar-CH_3_); ^13^C-NMR (DMSO-*d_6_*) δ 188.74, 146.83, 129.96, 129.33, 128.76, 56.20, 24.71, 24.63, 21.51. MS (ESI) *m/z*: 273.2 [C_11_H_14_BrOS]^+^; Anal. calcd. for C_11_H_14_Br_2_OS: C, 37.31; H, 3.99. Found: C, 37.39; H, 4.13.

*Dimethyl-(a-bromo-4-nitrophenacyl)sulfonium bromide* (**3f**). Yield: 55%, mp 138–139 °C. ^1^H-NMR (DMSO-*d_6_*) δ 8.49 (d, *J* = 8.9 Hz, 2H, Ar-H), 8.30 (d, *J* = 8.8 Hz, 2H, Ar-H), 7.94 (s, 1H, CH), 3.07 (s, 3H, SCH_3_), 3.03 (s, 3H, SCH_3_); ^13^C-NMR (DMSO-*d_6_*) δ 190.73, 151.03, 136.27, 131.17, 124.34, 56.33, 24.75; MS (ESI) *m/z*: 304.2 [C_10_H_11_BrNO_3_S]^+^; Anal. calcd. for C_10_H_11_Br_2_NO_3_S: C, 31.19; H, 2.88. Found: C, 31.25; H, 2.71.

*Dimethyl-(a-bromo-4-chlorophenacyl)sulfonium bromide* (**3g**). Yield: 57%, mp 152–153 °C. ^1^H-NMR (DMSO-*d_6_*) δ 8.09 (d, *J* = 8.5 Hz, 2H, Ar-H), 7.83 (s, 1H, CH), 7.78 (d, *J* = 8.5 Hz, 2H, Ar-H), 3.06 (s, 3H, SCH_3_), 3.00 (s, 3H, SCH_3_); ^13^C-NMR (DMSO-d_6_) δ 188.23, 140.63, 131.61, 130.14, 129.60, 56.25, 24.65; MS (ESI) *m/z*: 293.2 [C_10_H_11_BrClOS]^+^; Anal. calcd. for C_10_H_11_Br_2_ClOS: C, 32.07; H, 2.96. Found: C, 32.15; H, 2.85.

*Dimethyl-(a-bromo-4-fluorophenacyl)sulfonium bromide* (**3h**). Yield: 48%, mp 141–143 °C. ^1^H-NMR (DMSO-*d_6_*) δ 8.17 (dd, *J* = 8.8, 5.4 Hz, 2H, Ar-H), 7.77 (s, 1H, CH), 7.54 (t, *J* = 8.8 Hz, 2H, Ar-H), 3.05 (s, 3H), 2.98 (s, 3H); ^13^C-NMR (DMSO-*d_6_*) δ 190.12, 166.05 (d, *J* = 254.5 Hz), 131.96 (d, *J* = 10.0 Hz), 130.83 (d, *J* = 2.8 Hz), 116.46 (d, *J* = 22.3 Hz), 52.82, 24.71; MS (ESI) *m/z*: 277.1 [C_10_H_11_BrFOS]^+^; Anal. calcd. for C_10_H_11_Br_2_FOS: C, 33.54; H, 3.10. Found: C, 33.46; H, 3.21.

## 4. Conclusions

In conclusion, the combination DMSO and aqueous HBr has been utilized here for the efficient synthesis of dimethyl aryl acylsulfonium bromides from aryl methyl ketones under mild reaction conditions. In some cases, α-bromoacylsulfonium salt products were obtained.
